# Lower Performance in the Six-Minute Walk Test in Obese Youth With Cardiometabolic Risk Clustering

**DOI:** 10.3389/fendo.2018.00701

**Published:** 2018-11-27

**Authors:** Giuliana Valerio, Maria Rosaria Licenziati, Paola Tortorelli, Lidia Federica Calandriello, Paola Alicante, Luca Scalfi

**Affiliations:** ^1^Department of Movement Sciences and Wellbeing, University of Naples “Parthenope,” Naples, Italy; ^2^Obesity and Endocrine Disease Unit, Department of Neuroscience, Santobono-Pausilipon Children's Hospital, Naples, Italy; ^3^Department of Public Health, School of Medicine, University of Naples Federico II, Naples, Italy

**Keywords:** aerobic fitness, cardiometabolic risk, muscular fitness, obesity, six-minute walk test, youth

## Abstract

**Background:** Physical fitness is an important index of health. Our aim was to assess whether cardiorespiratory and/or musculoskeletal components of physical fitness were associated with cardiometabolic risk clustering in obese youth, using adapted and validated field tests.

**Methods:** We evaluated 252 children and adolescents (132 males, 120 females), mean age 10.9 ± 1.9 years with primary obesity. All subjects performed the six-minute walk test (6MWT) for assessing aerobic fitness, the standing broad jump, and the 30 s-chair stand tests for lower-body muscular strength, and the handgrip test for upper body isometric strength. Cardiometabolic risk (CMR) clustering was defined as having two or more of the following risk factors: high SBP and/or DBP, impaired fasting glucose, high triglycerides (TGs), and low HDL-Cholesterol.

**Results:** CMR clustering was found in 44 (17.5%) obese youth. Youth with CMR clustering had a lower cardiorespiratory fitness, as assessed by 6MWT, compared to those without CMR clustering. On the contrary, no difference was found with respect to musculoskeletal fitness. The six-minute walk (6MW) distance was negatively associated with sedentary time, controlling for age and height. CMR factors clustering was significantly associated with BMI standard deviation score (SDS) and negatively with 6MW distance: for each 10-m increase in the 6MW distance, a reduction of about 9% in the prevalence of CMR clustering was expected.

**Conclusions:** A lower performance in the 6MWT may be considered as an additional trait of CMR clustering in obese youth. The 6MWT may represent a valuable, simple and low cost test to estimate the cardiorespiratory fitness in youth with obesity.

## Introduction

Physical fitness, an index of body function with respect to the performance of daily physical activity and/or physical exercise, may be considered one of the most important health markers in children and adolescents (1), as well as, a predictor of cardiometabolic risk (CMR) in young adulthood (2, 3). In addition to cardiorespiratory fitness, which has long been associated with health outcomes, musculoskeletal fitness is also recognized as a crucial component in maintaining overall health in youth (4). Both low aerobic and musculoskeletal fitness have been related to the traditional CMR factors, such as abdominal adiposity, insulin resistance, and dyslipidemia (5–8) in the first two decades of life.

It is well-known that physical fitness is impaired in obese children and adolescents, and represents the main reason for their low participation in physical activities (9, 10). On the other hand, as far as we know, few papers have assessed whether physical fitness may be related to CMR clustering in obese children, with contrasting results (11, 12). In these studies, cardiorespiratory fitness was assessed during cycling graded exhaustive test, hence the results might have been undermined, since obese youth rarely engage in physical activity of high intensity (13). Therefore, the aim of this study was to assess whether physical fitness was associated with CMR clustering in obese youth, using adapted and validated field tests to assess the cardiorespiratory and/or musculoskeletal components of physical fitness.

## Materials and methods

### Subjects

In this cross-sectional study 252 children and adolescents (132 males, 52.4%, mean age 10.9 ± 1.9 years) with primary obesity were consecutively recruited at the Childhood Obesity clinic of Santobono-Annunziata Hospital in Naples, Italy. Participants had been referred to the hospital for assessment of health status and implementation of a weight loss program. The inclusion criteria were: Caucasian origin, and chronological age between 8 and 16 years. The exclusion criteria were the presence of any specific genetic or endocrine pathologic process which may cause obesity, personal history of diabetes mellitus, hypertension, or low-density lipoprotein-cholesterol (LDL-C) ≥190 mg/dL. The study took place from 1st April 2017 to 31th December 2017. The protocol was approved by the Ethics Committee of the Cardarelli-Santobono Hospital (reference number 233/28.03.2017). Written informed consent for all procedures was obtained from the youth and/or their parents or legal guardians in accordance with the Declaration of Helsinki.

### Anthropometric measurements

Height, weight, and waist circumference (WC) were measured by the same investigator with the children wearing only light clothes and no shoes. Following standard procedures, height was measured to the nearest 0.1 cm with a wall-mounted stadiometer, while weight was determined to the nearest 0.1 kg on a medical scale. WC was measured with the child in a standing position with a flexible tape taken midway between the 10th rib and the iliac crest. The body mass index (BMI) [weight (kg)/height (m^2^)] was calculated. Since height and BMI are age- and gender-related, these parameters were transformed into standard deviation scores (SDS), based upon the established Italian BMI normative curves (14).

### Cardiometabolic risk factors

Blood pressure was measured using aneroid sphygmomanometers with cuffs of appropriate size, according to standard procedures. Briefly, blood pressure was measured in the right arm in sitting position after 5-min resting, using the auscultatory method; Korotkoff 1 was used for systolic blood pressure (SBP) and Korotkoff 5 for diastolic blood pressure (DBP) as recommended (15).

Plasma glucose, total cholesterol, triglycerides (TGs), and high-density lipoprotein-cholesterol (HDL-C) were measured in fasting blood samples using standardized methods.

### Definitions

Obesity was defined according to BMI >95th percentile of the established Italian BMI normative curves (14). Cardiometabolic risk (CMR) clustering was defined as having two or more of the following listed risk factors: SBP and/or DBP ≥95th percentile for age, sex, and height (15), Fasting plasma glucose ≥100 mg/dL,TG≥100 mg/dL in children, and ≥130 mg/dL in adolescents, HDL-C < 40 mg/dL (16).

### Physical activity and sedentary time assessment

The study included a questionnaire assessment by interview regarding some behavioral issues of youth, i.e., sports participation in the previous 6 months (h/week) and sedentary time (h/day), as the sum of the daily hours spent in television viewing, videogames, and surfing on computer.

### Physical fitness assessment

Cardiorespiratory fitness and musculoskeletal fitness were evaluated by the same investigator (PT), who was trained in physical fitness assessment. Each test was conducted individually; participants wore their usual comfortable footwear.

### Six-minute walk test (6MWT)

The six-minute walk test (6MWT) was performed indoors along a flat, straight walkway in accordance with the American Thoracic Society guidelines (17). The walking course length measured 20 m. The length of the corridor was marked every 3 m with a brightly colored tape. Cones were placed at either end of the walking course to indicate the beginning and end points. Additionally, the starting line, which marked the beginning and end of each lap, was marked on the floor using brightly colored tape. Instructions and demonstrations were given to each child. Participants were tested individually. They were informed that the purpose of the test was to find out how far they could walk in 6-min and were instructed to walk the longest distance possible at their own pace during the allotted time. Hopping, skipping, running, and jumping were not allowed during the test. Only the standardized phrases for encouragement (e.g., “keep going,” “you are doing well”) and announcement of time remaining were given to the participants.

### Lower-body muscular strength

The lower-body strength was assessed by the standing broad jump and the 30 s-chair stand tests. For the standing broad jump, the participant stood behind the starting line and was instructed to push off vigorously and jump as far as possible. The participant had to land with the feet together and to stay upright. The distance in centimeter was measured from the take-off line to the point where the back of the heel nearest to the take-off line lands on the ground. A further attempt was allowed if the subject fell backward or touched the mat with another part of the body. The test was repeated twice, and the best score was considered (18).

For the chair stand test an armless, height-adjustable chair was used. The participant started in a seated position with the back against the backrest of the chair and knees in 90° of flexion. The participant had to stand up and sit down as quickly as possible with arms folded across chest, paying attention to stand up until knees were fully straightened and lean back against the backrest when sitting down. The maximum number of full stands in 30 s was retained (19).

### Upper muscular strength

The upper body isometric strength was assessed by the handgrip test, using a hand dynamometer with adjustable grip (TKK 5101 Grip D; Takey, Tokio Japan). The participant held the instrument, with the elbow in full extension and avoiding contacting of any other part of the body with the dynamometer, except the hand being measured, then squeezed gradually and continuously for at least 5 s. The test was performed three times (alternately with both hands), allowing 15 s rest between measures. The best result (expressed in kg, accurate to 0.1 kg) in the dominant hand was retained (18).

### Statistical analysis

Statistical analysis was carried out using the Statistical Package of Social Sciences (SPSS, Windows release 24.0; Chicago, IL, USA). Results are presented as mean ± Standard Deviation, unless otherwise stated, with statistical significance set at *p* ≤ 0.05. The sample size necessary to achieve a *P*-value = 0.02 with a correlation coefficient of 0.2 and power of 80% was 247.

The Kolmogorov-Smirnov goodness-of-fit test was applied for determining whether sample data likely derive from a normally distributed population. Variables not normally distributed were logarithmically transformed. However, for clarity of interpretation, results are expressed as untransformed values. The independent sample *t*-test was used to compare the means of continuous variables, while contingency table analyses were used for categorical variables. Differences in physical fitness tests between groups with and without CMR clustering were tested by univariate general linear model, controlling for gender, age, height, and BMI-SDS. Pearson correlation coefficients were performed to examine the association between the six-minute walk (6MW) distance and the variables of interest. The variables significantly associated with the 6MW were entered in a partial correlation analysis. Binary logistic regression analysis was performed to evaluate the associations of CMR clustering with 6MW distance in decameter, gender, age, and BMI-SDS and expressed as odds ratios (OR) and 95% confidence intervals (CI).

## Results

Anthropometric, lifestyle, cardiometabolic, and physical fitness features of the study population are shown in Table 1 according to gender. Females were less sedentary, but spent less time on sports, showed higher TG levels and lower values of musculoskeletal strength, i.e., standing broad jump and handgrip test, than males. Instead, the 6MW distance did not differ between genders.

**Table 1:** Anthropometric, lifestyle, cardiometabolic, and physical fitness features of the study population according to gender.

	**Males**	**Females**	***P***
Number	132	120
Age, years	11.1 ± 1.8	10.8 ± 1.9	0.11
Height, cm	149.0 ± 12.3	146.3 ± 10.4	0.07
Weight, kg	68.9 ± 18.1	66.3 ± 16.5	0.26
BMI, kg/m^2^	30.6 ± 5.1	30.5 ± 4.3	0.95
BMI-SDS	2.3 ± 0.5	2.3 ± 0.4	0.24
Waist, cm	100.9 ± 11.3	99.6 ± 11.6	0.33
**LIFESTYLE**			
Sports participation, *N* (%)	61 (46.2)	49 (40.1)	0.39
Sports participation, h/week^*^	3.8 ± 1.8	2.8 ± 1.3	< 0.01
Sedentary time, h/day	4.8 ± 2.1	4.1 ± 2.2	< 0.01
**CARDIOMETABOLIC RISK FACTORS**			
Systolic blood pressure, mmHg	106.7 ± 13.1	106.7 ± 12.4	0.91
Diastolic blood pressure, mmHg	63.7 ± 8.5	64.5 ± 7.9	0.22
Glucose, mg/dL	90.9 ± 7.9	90.6 ± 10.9	0.71
Triglycerides, mg/dL	88.4 ± 41.4	102.9 ± 50.5	< 0.01
Total cholesterol, mg/dL	155.7 ± 32.7	152.1 ± 30.7	0.47
HDL cholesterol, mg/dL	49.5 ± 12.5	46.4 ± 10.6	0.06
**PHYSICAL FITNESS**			
Six-minute walk distance, m	460.6 ± 44.8	454.4 ± 45.8	0.34
Standing broad jump, cm	92.9 ± 20.2	80.5 ± 18.6	< 0.01
Chair stand, no. of stands	17.2 ± 3.2	17.2 ± 3.3	0.91
Handgrip strength, kg	19.1 ± 5.4	17.8 ± 5.1	< 0.05

*Data are expressed as mean ± standard deviation or number (percentage)*.

* *These data are referred only to sports participants*.

Forty-four (17.5%) youth presented CMR clustering. Table 2 summarizes the anthropometric, lifestyle, and cardiometabolic features of the study population, according to CMR clustering. Besides the values of CMR factors, which were consistent with the study design, the two groups differed in gender distribution (*P* = 0.04) and BMI-SDS (*P* = 0.03), while no difference was found with regard to sports participation and sedentary time.

**Table 2:** Anthropometric, lifestyle, and cardiometabolic features of the study population according to cardiometabolic risk clustering.

	**Cardiometabolic risk clustering**	

	**Absent**	**Present**	***P***
Number	208	44
Males/females, *N* (%)	115/93 (55.3/44.7)	17/27 (38.6/61.4)	0.04
Age, years	11.0 ± 1.9	10.5 ± 1.8	0.09
Height, cm	147.8 ± 11.5	147.4 ± 11.6	0.82
Weight, kg	66.9 ± 16.1	70.8 ± 22.2	0.54
BMI, kg/m^2^	30.3 ± 4.4	31.9 ± 5.9	0.09
BMI-SDS	2.3 ± 0.4	2.5 ± 0.5	0.03
Waist, cm	99.9 ± 11.3	102.3 ± 11.9	0.73
**LIFESTYLE**			
Sports participation, *N* (%)	92 (44.2)	18 (40.9)	0.69
Physical activity, h/week^*^	3.5 ± 1.7	2.9 ± 1.4	0.12
Sedentary time, h/day	4.4 ± 2.1	4.7 ± 2.4	0.67
**CARDIOMETABOLIC RISK FACTORS**			
Systolic blood pressure, mmHg	105.6 ± 12.3	111.9 ± 13.9	< 0.01
Diastolic blood pressure, mmHg	63.4 ± 7.8	67.4 ± 9.5	< 0.01
Glucose, mg/dl	90.4 ± 6.7	92.5 ± 17.5	< 0.01
Triglycerides, mg/dl	83.9 ± 34.4	148.1 ± 58.4	< 0.01
Total cholesterol, mg/dl	151.5 ± 31.1	165.7 ± 32.4	< 0.01
HDL cholesterol, mg/dl	49.8 ± 11.2	39.4 ± 10.3	< 0.01

*Data are expressed as mean ± standard deviation or number (percentage)*.

* *These data are referred only to sports participants*.

Table 3 shows the estimated marginal mean values of the physical fitness tests in the two groups with or without CMR clustering. Data were adjusted for age, gender, height, and BMI-SDS. Youth with CMR clustering exhibited a significant worse performance in the 6MWT, while no difference was found regarding to lower and upper body strength.

**Table 3:** Estimated marginal mean values of the physical fitness tests of the study population according to cardiometabolic risk clustering.

	**Cardiometabolic risk clustering**	

	**Absent**	**Present**	***P***
Six-minute walk distance, m	460.9 ± 3.1 (454.8–466.9)	442.4 ± 6.8 (428.9–455.9)	< 0.01
Standing broad jump, cm	86.7 ± 1.3 (84.1–89.3)	88.9 ± 2.9 (82.9–94.4)	0.48
Chair stand, no. of stands	17.2 ± 0.2 (16.8–17.7)	17.1 ± 0.5 (16.1–18.1)	0.74
Handgrip strength, kg	18.5 ± 0.3 (17.9–19.1)	18.3 ± 0.6 (17.1–19.5)	0.72

*Data are expressed as estimated marginal means ± SE (95% CI) adjusted for age, gender, height, and BMI-SDS*.

**Figure 1:**
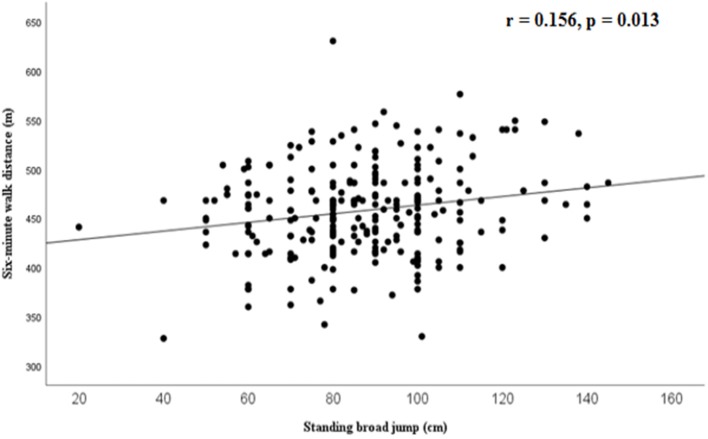
Correlation between six-minute walk distance and standing broad jump.

**Figure 2:**
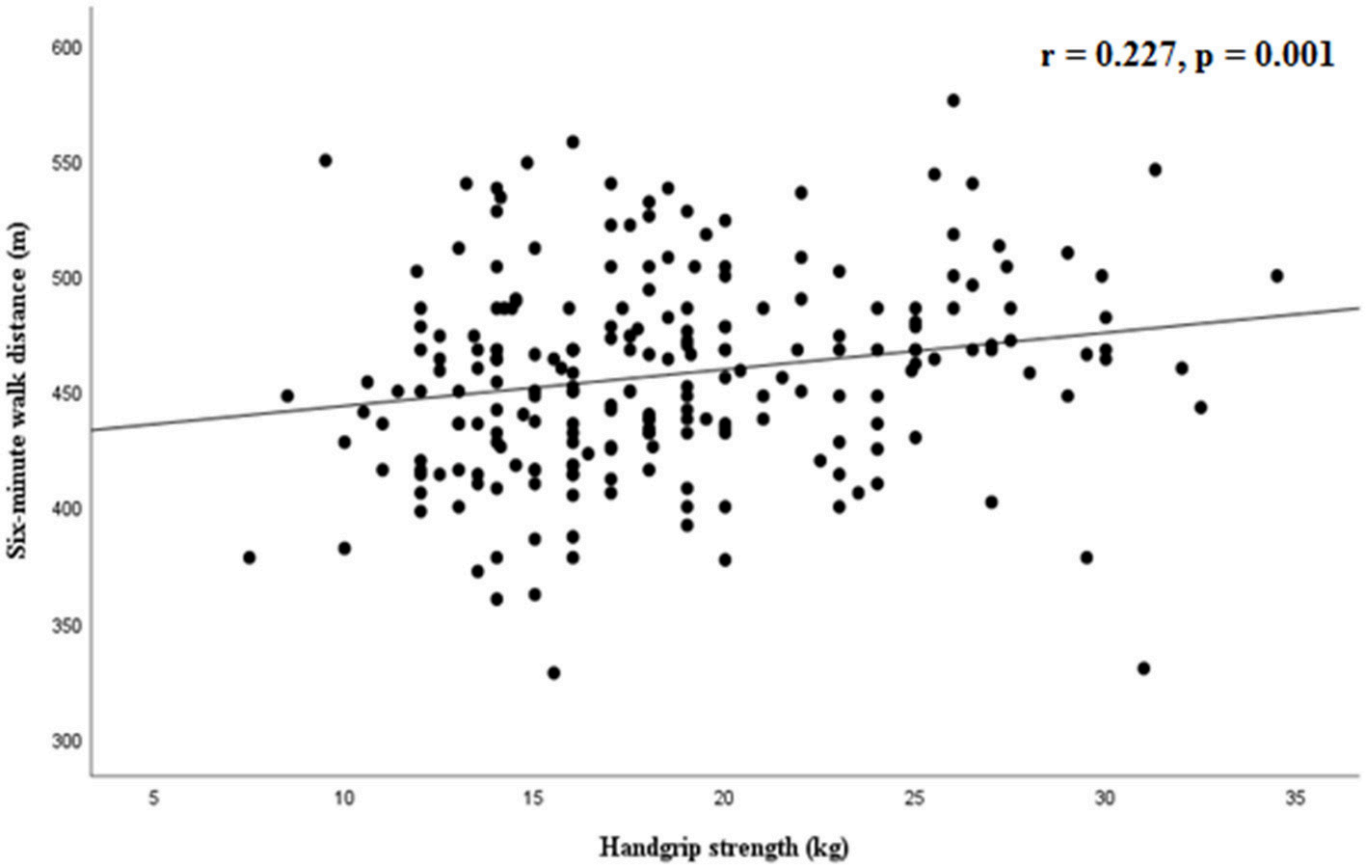
Correlation between six-minute walk distance and handgrip strength.

**Figure 3:**
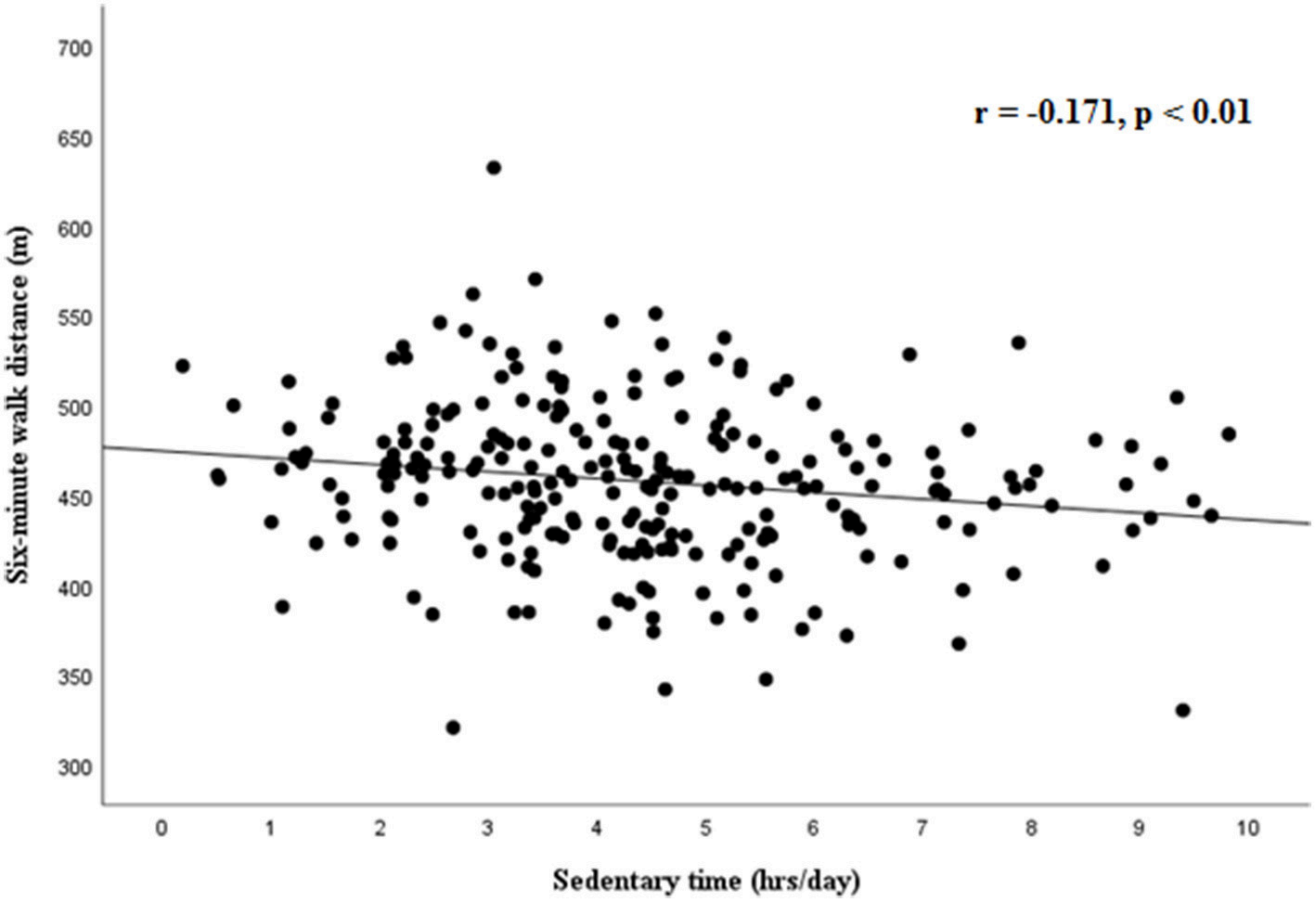
Partial correlation between six-minute walk distance and sedentary time, controlling for age and height.

The 6MW distance was significantly correlated with standing broad jump (*r* = 0.156, *p* = 0.013, Figure 1) and handgrip strength (*r* = 0.227, *p* = 0.001, Figure 2), while a weaker correlation was found with chair test (*r* = 0.122, *p* = 0.055). Furthermore, the 6MW distance was positively correlated with age (*r* = 0.204, *p* < 0.01), height (*r* = 0.232, *p* < 0.001), and negatively with sedentary time (*r* = −0.146, *p* = 0.02), while no significant correlation was found with BMI-SDS (*r* = 0.035) and weekly hours of sports participation (*r* = 0.052). Partial correlation analysis confirmed that the 6MW distance was negatively related to sedentary time, controlling for age and height (*r* = −0.171, *p* < 0.01) (Figure 3).

Lastly, logistic regression analysis showed that CMR clustering was positively associated with BMI-SDS (OR 3,230, 95% CI 1.470–7.096, *p* < 0.01) and negatively with 6MW distance (for a 10-min increase: OR 0.911, 95% CI 0.840–0.988), while no significant association was found with gender (reference males, OR 1.769, 95% CI 0.887–3.529) and age (OR 0.823, 95% CI 0.673–1.008).

## Discussion

The present study adds to the current knowledge that obese youth with CMR clustering have a decreased cardiorespiratory fitness, as assessed by 6MWT, compared to those without CMR clustering. On the contrary, no difference was found with respect to musculoskeletal fitness.

Due to the strict relationships between physical activity, body weight and health, the assessment of physical fitness plays a central role in the management of obesity. Numerous studies have examined the impact of weight status on health-related physical fitness, utilizing those field-based methods, which appear more suitable in obese children than laboratory-based methods. Worse performance in weight bearing tasks, such as walking and running, and impaired cardiorespiratory fitness have been consistently reported in obese youth compared with non-obese youth (20). In contrast, the evidence of impaired muscle strength is still controversial (21).

The role of fitness in the metabolically unhealthy obese phenotype has been reviewed by Ortega et al. (22), suggesting that a lower cardiorespiratory fitness should be considered a characteristic of the metabolically unhealthy phenotype, at least in adult populations. As far as we know, few papers have assessed the physical fitness in obese children with CMR clustering, with contrasting results. While some studies suggest that high cardiorespiratory fitness levels confer significant protection from CMR clustering in youth and can modify the impact that BMI has on the clustered CMR risk factors in children (11), other studies reported no association at all (12, 23). Noteworthy, these studies have measured cardiorespiratory fitness in obese children by incremental tests on cycle ergometer (24). However, the use of this technique is limited because of high cost and requirement of sophisticated equipment and specialized medical personnel.

Recently, an integrative review recommended the 6MW test as the most suitable field test for monitoring physical fitness in obese youth, since it fulfilled several measurement properties with respect to the numerous other field tests of physical function assessed in children with obesity (25). Since it is supposed to be better tolerated by individuals with physical limitation, this test may better reflect the functional status of individuals with excess body weight.

In a large clinical sample of children and adolescents with obesity our results demonstrated that the 6MW distance was significantly lower in obese youth with CMR clustering, controlling for gender, age, and BMI-SDS. We found difference of about 18 min in the 6MW distance between youth with and without CMR clustering. This small effect size is reasonable and expected, since our analysis was restricted only to youth with moderate/severe obesity. Logistic analysis showed that for each 10-m increase in the 6MW distance, a reduction of about 9% in the prevalence of CMR clustering was expected.

Lacking comparable studies in obese youth, we report two studies in Canadian and Moroccan women with obesity, that demonstrated greater 6MW distance in metabolically healthy obese than non-metabolically healthy obese individuals (26, 27). On the contrary, our results are in contrast with the unique pediatric study by Cadenas-Sanchez et al. (28), who used a different cardiorespiratory field test, i.e., the 20 m shuttle run test, without demonstrating any consistent difference between metabolically healthy and non-metabolically healthy adolescents with overweight or obesity. At variance with the 6MWT, the 20 m shuttle run test is a multistage test that explores speed and agility and is widely employed in athletes of all levels (29). Furthermore, we found no association between sports participation or sedentary time and CMR clustering, at variance with the study by Cadenas-Sanchez et al. (28). The different methods employed to assess the lifestyle behaviors, i.e., a questionnaire in our study and accelerometry in the Cadenas-Sanchez et al's study, might explain this divergence.

The well-known association between 6MW distance, age, and height (30) has been confirmed also in our sample of obese youth by simple correlation analyses. Instead, no difference in the 6MW distance was found between genders, reasonably because they had similar height and BMI-SDS. Interestingly, among the lifestyle behaviors, lower 6MW distance was associated with sedentary time, controlling for age and height. However, given the cross-sectional design of the study, a causal relationship cannot be demonstrated. Walking capacity was significantly associated with muscle strength, in particular with the handgrip test, which is considered a good index for overall muscle strength. Both aerobic fitness and muscular strength are domains of health-related fitness, representing the motor capacity of the subject. Our results suggest that individuals who possess higher strength tend also to perform better in 6MWT.This is the first evidence in obese children, while a similar association was reported in adults (31).

Although it has been reported that muscle strength in youth is a powerful predictor of better insulin sensitivity in children and adolescents (7), there is still an inconclusive evidence for a relationship between muscular strength and CMR risk factors in youth (4, 32).

Therefore, we were also interested to assess whether musculoskeletal fitness, i.e., lower and upper body muscular strength, was associated with CMR clustering in obese youth. For this purpose, we used the broad jump and the handgrip tests as suggested by the Alpha fitness battery for young people (18). Considering that obese youth are limited in basic activities, such as moving between seated and upright postures (33), we also included the 30 s chair stand test, derived from the Senior fitness battery (19).

In agreement with Cadenas-Sanchez et al. (28), who also measured the broad jump and the handgrip tests in their population, we found no significant association between musculoskeletal fitness and CMR clustering.

A few limitations should be acknowledged. First, the cross-sectional design of the study, which does not imply a causal relationship between physical fitness and CMR clustering. Moreover, the assessment of sports participation as the only index of physical activity might have contributed to losing important associations between physical activity and CMR clustering.

The strength of the study is the peculiarity of the physical fitness components examined (aerobic, fitness, lower-, and upper-body strength), which allowed us to make comparisons between the main components of the health related physical fitness and CMR clustering. Furthermore, measurements of physical fitness were performed by the same investigator and fulfilled well-defined protocols; therefore, any bias due to inter-observer variability was excluded.

In conclusion, our study allowed us to identify the 6MWT as a useful test of physical fitness in youth with obesity in a clinical setting. A lower performance in the 6MWT may be considered as an additional trait of CMR clustering in obese youth, while musculoskeletal fitness was not a contributory factor. The 6MWT may represent a valuable, simple and low cost test to estimate the cardiorespiratory fitness in youth with obesity.

## Author contributions

GV conceived and designed the study, performed statistical analysis and interpretation of the data, and wrote the draft. ML took part in designing the study, provided data for the study, and revised the manuscript critically. PT, LC, and PA provided data for the study and made their contribution in revising the manuscript. LS gave a substantial contribution to interpretation of the data and revised the manuscript critically. All of the authors read and approved the final version of the manuscript.

### Conflict of interest statement

The authors declare that the research was conducted in the absence of any commercial or financial relationships that could be construed as a potential conflict of interest.

## Abbreviations

BMI: body mass index
CMR: cardiometabolic risk
DBP: diastolic blood pressure
HDL-C: high-density lipoprotein-cholesterol
6MWT: six-minute walk test
SDS: standard deviation scores
SBP: systolic blood pressure
TGs: triglycerides
WC: waist circumference.

## References

[1] Ortega FB Ruiz JR Castillo MJ Sjöström M. Physical fitness in childhood and adolescence: a powerful marker of health. Int J Obes. (2008) 32:1–11. 10.1038/sj.ijo.0803774 18043605

[2] Andersen LB Hasselstrøm H Grønfeldt V Hansen SE Karsten F. The relationship between physical fitness and clustered risk, and tracking of clustered risk from adolescence to young adulthood: eight years follow-up in the Danish Youth and Sport Study. Int J Behav Nutr Phys Act. (2004) 1:6. 10.1186/1479-5868-1-6 15169561 PMC416568

[3] Hasselstrøm H Hansen SE Froberg K Andersen LB Physical fitness and physical activity during adolescence as predictors of cardiovascular disease risk in young adulthood. Danish Youth and Sports Study. An eight-year follow-up study. Int J Sports Med. (2002) 23 (Suppl. 1):S27–31. 10.1055/s-2002-28458 12012259

[4] Ruiz JR Castro-Piñero J Artero EG Ortega FB Sjöström M Suni J. Predictive validity of health-related fitness in youth: a systematic review. Br J Sports Med. (2009) 43:909–23. 10.1136/bjsm.2008.056499 19158130

[5] Froberg K Andersen LB. Mini review: physical activity and fitness and its relations to cardiovascular disease risk factors in children. Int J Obes. (2005) 29 (Suppl. 2):S34–9. 10.1038/sj.ijo.0803096 16385750

[6] Zaqout M Michels N Bammann K Ahrens W Sprengeler O Molnar D. Influence of physical fitness on cardio-metabolic risk factors in European children. The IDEFICS study. Int J Obes. (2016) 40:1119–25. 10.1038/ijo.2016.22 26857382

[7] Benson AC Torode ME Singh MA. Muscular strength and cardiorespiratory fitness is associated with higher insulin sensitivity in children and adolescents. Int J Pediatr Obes. (2006) 1:222–31. 10.1080/17477160600962864 17907329

[8] Smith JJ Eather N Morgan PJ Plotnikoff RC Faigenbaum AD Lubans DR. The health benefits of muscular fitness for children and adolescents: a systematic review and meta-analysis. Sports Med. (2014) 44:1209–23. 10.1007/s40279-014-0196-4 24788950

[9] Norman AC Drinkard B McDuffie JR Ghorbani S Yanoff LB Yanovski JA. Influence of excess adiposity on exercise fitness and performance in overweight children and adolescents. Pediatrics (2005) 115:e690–6. 10.1542/peds.2004-1543 15930197 PMC1350764

[10] Valerio G Gallarato V D'Amico O Sticco M Tortorelli P Zito E. Perceived difficulty with physical tasks, lifestyle, and physical performance in obese children. Biomed Res Int. (2014) 2014:735764. 10.1155/2014/735764 25105139 PMC4106089

[11] DuBose KD Eisenmann JC Donnelly JE. Aerobic fitness attenuates the metabolic syndrome score in normal-weight, at-risk-for-overweight, and overweight children. Pediatrics (2007) 120:e1262–8. 10.1542/peds.2007-0443 17974719

[12] Shaibi GQ Cruz ML Ball GD Weigensberg MJ Kobaissi HA Salem GJ. Cardiovascular fitness and the metabolic syndrome in overweight Latino youths. Med Sci Sports Exerc. (2005) 37:922–8. 10.1029/01.mss.0000170472.75214.53 15947715

[13] Keane E Li X Harrington JM Fitzgerald AP Perry IJ Kearney PM. Physical activity, sedentary behavior and the risk of overweight and obesity in school-aged children. Pediatr Exerc Sci. (2017) 29:408–18. 10.1123/pes.2016-0234 28388271

[14] Cacciari E Milani S Balsamo A Spada E Bona G Cavallo L. Italian cross-sectional growth charts for height, weight and BMI (2 to 20 yr). J Endocrinol Invest. (2006) 29:581–93. 10.1007/BF03344156 16957405

[15] Lurbe E Agabiti-Rosei E Cruickshank JK Dominiczak A Erdine S Hirth A. European Society of Hypertension guidelines for the management of high blood pressure in children and adolescents. J Hypertens. (2016) 34:1887–920. 10.1097/HJH.0000000000001039 27467768

[16] Expert Panel on Integrated Guidelines for Cardiovascular Health and Risk Reduction in Children and Adolescents; National Heart Lung and Blood Institute Expert panel on integrated guidelines for cardiovascular health and risk reduction in children and adolescents: summary report. Pediatrics (2011) 128(Suppl. 5):S213–56. 10.1542/peds.2009-2107C 22084329 PMC4536582

[17] ATS Committee on Proficiency Standards for Clinical Pulmonary Function Laboratories ATS statement: guidelines for the six-minute walk test. Am J Respir Crit Care Med. (2002) 166:111–17. 10.1164/ajrccm.166.1.at1102 12091180

[18] Ruiz JR Castro-Pinero J Espana-Romero V Artero EG Ortega FB Cuenca MM. Field-based fitness assessment in young people: the ALPHA health-related fitness test battery for children and adolescents. Br J Sports Med. (2011) 45:518e24. 10.1136/bjsm.2010.075341 20961915

[19] Rikli RE Jones CJ Senior Fitness Test Manual. Champaign, IL: Human Kinetics (2001).

[20] Tsiros MD Coates AM Howe PR Grimshaw PN Buckley JD Obesity: the new childhood disability? Obes Rev. (2011) 12:26–36. 10.1111/j.1467-789X.2009.00706.x 20070542

[21] Thivel D Ring-Dimitriou S Weghuber D Frelut ML O'Malley G. Muscle strength and fitness in pediatric obesity: a systematic review from the European Childhood Obesity Group. Obes Facts (2016) 9:52–63. 10.1159/000443687 26901423 PMC5644904

[22] Ortega FB Cadenas-Sánchez C Sui S Blair SN Lavie CJ. Role of fitness in the metabolically healthy but obese phenotype: a review and update. Prog Cardiovasc Dis. (2015) 58:76–86. 10.1016/j.pcad.2015.05.001 25959452

[23] Sénéchal M Wicklow B Wittmeier K Hay J MacIntosh AC Eskicioglu P. Cardiorespiratory fitness and adiposity in metabolically healthy overweight and obese youth. Pediatrics (2013) 132:e85–92. 10.1542/peds.2013-0296 23796736

[24] Owens S Gutin B. Exercise testing of the child with obesity. Pediatr Cardiol. (1999) 20:79–83. 10.1007/s002469900405 9861087

[25] Mahaffey R Morrison SC Stephensen D Drechsler WI. Clinical outcome measures for monitoring physical function in pediatric obesity: an integrative review. Obesity (2016) 24:993–1017. 10.1002/oby.21468 27062537

[26] Bouchard DR Langlois MF Brochu M Dionne IJ Baillargeon JP. Metabolically healthy obese women and functional capacity. Metab Syndr Relat Disord. (2011) 9:225–9. 10.1089/met.2010.0101 21361821

[27] Aparicio VA Carbonell-Baeza A Senhaji M Martín S Camiletti-Moirón D Aranda P. Usefulness of fitness testing to establish metabolic syndrome in perimenopausal Moroccan women. Eur J Cardiovasc Nurs. (2013) 13:524–31. 10.1177/1474515113516395 24322546

[28] Cadenas-Sanchez C Ruiz JR Labayen I Huybrechts I Manios Y González-Gross M. Prevalence of metabolically healthy but overweight/obese phenotype and its association with sedentary time, physical activity, and fitness. J Adolesc Health (2017) 61:107–114. 10.1016/j.jadohealth.2017.01.018 28363717

[29] Paradisis GP Zacharogiannis E Mandila D Smirtiotou A Argeitaki P Cooke CB. Multi-stage 20-m shuttle run fitness test, maximal oxygen uptake and velocity at maximal oxygen uptake. J Hum Kinet. (2014) 41:81–7. 10.2478/hukin-2014-0035 25114734 PMC4120467

[30] Geiger R Strasak A Treml B Gasser K Kleinsasser A Fischer V. Six-minute walk test in children and adolescents. J Pediatr. (2007) 150:395–9. 10.1016/j.jpeds.2006.12.052 17382117

[31] Zhang Q Lu H Pan S Lin Y Zhou K Wang L 6MWT performance and its correlations with VO2 and handgrip strength in home-dwelling mid-aged and older Chinese. Int J Environ Res Public Health (2017) 14:E473 10.3390/ijerph14050473 28468260 PMC5451924

[32] Tsiros MD Buckley JD Howe PR Olds T Walkley J Taylor L. Day-to-day physical functioning and disability in obese 10- to 13-year-olds. Pediatr Obes. (2013) 8:31–41. 10.1111/j.2047-6310.2012.00083.x 22962042

[33] Peterson MD Saltarelli WA Visich PS Gordon PM. Strength capacity and cardiometabolic risk clustering in adolescents. Pediatrics (2014) 133:e896–903. 10.1542/peds.2013-3169 24685949 PMC4530295

